# European Psychiatric Association–European Academy of Neurology statement on post-COVID syndrome

**DOI:** 10.1192/j.eurpsy.2022.2317

**Published:** 2022-09-08

**Authors:** Claudio L. A. Bassetti, Raimund Helbok, Kristina Adorjan, Peter Falkai

**Affiliations:** 1 Department of Neurology, Inselspital, University of Bern, Bern, Switzerland; 2 European Academy of Neurology (EAN); 3 Department of Neurology, Neurocritical Care Unit, Medical University of Innsbruck, Innsbruck, Austria; 4 Department of Psychiatry and Psychotherapy, LMU University Hospital, Munich, Germany; 5 European Psychiatric Association (EPA)

## Abstract

We aimed to determine the role of the European Psychiatric Association (EPA) and the European Academy of Neurology (EAN) in the management of post-COVID conditions. This is a joint statement from the EAN and the EPA on post-COVID. It is published in the official journals of the two associations, the *European Journal of Neurology and European Psychiatry*.

## Introduction

Severe acute respiratory syndrome coronavirus 2 (SARS-CoV-2) has rapidly emerged as a pandemic and caused morbidity and mortality to an inconceivable extent globally [1]. Emergence of different variants resulted in multiple waves of coronavirus disease-2019 (COVID-19) and has massively affected the world’s health and economy. As of July 2022, 565 million people have been infected with SARS-CoV-2, and 6.3 million died [2]. Acute COVID-19 has rapidly been recognized as a multiorgan disease reaching far beyond pulmonary symptoms and signs. These include neurologic, psychiatric, cardiac, and gastrointestinal manifestations among others. Soon, the World Federation of Neurology appealed to national and regional neurological associations to create databases for international neuroepidemiological collaboration [3]. Among others, the European Academy of Neurology (EAN) implemented an international registry to study neurological manifestations and long-term outcomes in COVID-19 patients (EAN NEuro-covid ReGistrY, ENERGY*)* [4], and furthermore signed a memorandum of understanding with the American Neurocritical Care Society to enlarge a global network [5]. Preexisting neurological diseases and new onset COVID-19 associated neurological manifestations have now been recognized as risk factors for a more severe disease and poor long term outcome [6, 7]. The European Psychiatric Association (EPA) established its “COVID-19 Resource Centre,” an online repository of high-quality COVID-19-related resources for both health professionals and the general public (https://www.europsy.net/covid-19-resource-centre/). The EPA and its network also implemented initiatives, such as surveys and events, to better understand the impact of COVID-19 on mental health and on the delivery of care. In addition, EPA supports regional projects such as the interdisciplinary and cross-sectoral care and research network (post-COVID^LMU^) at LMU University Hospital in Munich, Germany [8]. Finally, EPA and EAN jointly provide platforms for scientific exchange on COVID-19 and post-COVID in form of forums and symposia at congresses, conferences, and meetings.

Recognition of persistent symptoms and signs after recovery from initial COVID-19 illness have soon been recognized [9], and are currently referred to as “Long-COVID.” In fact, “Long-COVID” was created through social media [10], and is a time-based definition of unspecific symptoms and signs persisting beyond 4 weeks. The WHO recently recommended the use of the terminology of “post-COVID-19 condition,” which includes “individuals with a history of probable or confirmed SARS CoV-2 infection, usually 12 weeks from the onset of COVID-19 with symptoms and that last for at least 2 months and cannot be explained by an alternative diagnosis and has an impact on everyday functioning” [11]. There is currently a lack of robust evidence on the prevalence of these symptoms due to differences in reporting systems and inconsistency of study designs and definitions of symptoms, signs and diseases related to post-COVID conditions, which renders its global impact speculative. Recent estimates of the Office for National Statistics in Great Britain suggest that one in five patients with confirmed or suspected exhibits symptoms for a period of 5 weeks or longer and one in 10 for 12 weeks or longer [12]. These numbers rapidly change especially as the virus phenotype and infection rate varies. As of January 2022, an estimated 1.5 and 1.1 million people reporting symptoms beyond 4 and 12 weeks, respectively, are living in a private household in the UK (2.4 and 1.8% of the population).

Proposed mechanisms of persistent and new onset postinfectious disorders include immune dysregulation with persistent low-grade (neuro-)inflammation, immune dysregulation, autoimmunity, and viral persistence in various tissues [13]. Each of these hypotheses needs confirmation in larger scale studies and builds upon direct and indirect neuropathogenic effects of SARS-CoV-2 in the acute phase [14].

Long-term neurologic manifestations include fatigue, neurocognitive symptoms, sleep–wake disorders, dysautonomia, hyposmia, hypogeusia, and pain syndromes among others [13].

Fatigue was shown in a recent meta-analysis including 36 studies and a total of 9,944 participants to be the most common symptom (mean 52.8%; 95% Confidence Interval 19.9–84.4) [15]. The large 95% confidence intervals highlight the need for a more strict and homogenous definition of the post-COVID syndrome and its symptoms. The authors also suggest a better analysis in future studies of the longitudinal course of symtpoms, which appear to strongly fluctuate within the first year post-SARS-CoV-2 infection and even during the day.

Neurocognitive symptoms are commonly associated with impaired performance in neuropsychological testing and are expected sequelae among ICU survivors including those with COVID-19. Of note, early reports of frontotemporal FDG-PET hypometabolism [16] during acute COVID-19 have now been replicated in the subacute phase and associated with memory deficits and executive dysfunction [17]. Despite longitudinal recovery from cognitive deficits [18], the increased risk for neurological and psychiatric diagnoses in the 6 months after infection deserves attention [19]. These data derive from a large retrospective cohort of more than 250,000 survivors of COVID-19 reporting even a higher risk compared to propensity score-matched cohorts of influenza patients and those with other respiratory infections [19]. Interestingly, even patients with mild and moderate COVID-19 report poor concentration, lack of intellectual clarity and mental fatigue which is referred to as “brain fog” and is poorly captured by conventional testing including the MOCA test [20]. This and other consequences of COVID-19 lack of specificity and need a more precise definition. In general, neurological manifestations improve over time [6, 21]; however, long-term effects and new onset neurological diseases including autoimmune diseases and neurodegenerative diseases need close surveillance through national and international registries.

Non-pharmacological treatment strategies such as those recommended by the National Institute for Health and Excellence (NICE) are available for different symptoms of the post-COVID syndrome including self-management strategies, pacing, and multidisciplinary rehabilitation *(COVID-19 rapid guideline: managing the long-term effects of COVID-19 (magicapp.org)).* Pharmacological treatment for post-COVID neurological disease are based on proposed neuropathogenic mechanisms of SARS-CoV-2 infection including immunomodulation, IVIG/plasma-exchange for antibody/immune/cytokine-mediated para- and postinfectious diseases.

Specific guidelines however do not exist for post-COVID. Randomized controlled trials are needed to provide further treatment recommendations for subpopulation with distinct features. There will not be a single treatment alleviating all post-COVID symptoms. Researchers and clinicians are asked to link clinical phenotypes with biomarkers and define endpoints that can be used for clinical trials. A summary of ongoing clinical trials targeting several endpoints including neurocognition, anosmia, and headache has been recently published [22].

Among psychiatric disorders, anxiety, depression, insomnia, cognitive impairment, and post-traumatic stress disorder (PTSD) are the most common [23]. Relevant studies show a significantly increased incidence of mental disorders immediately following SARS-CoV-2 infection compared to unaffected populations [24]. A large-scale US study (*n* = 62,354) found an increased 3-month incidence of psychiatric diagnoses (18.1%) and first-episode psychiatric disorders (5.8%) after SARS-CoV-2 infection [25]. In a further study, an increased 6-month incidence of mental disorders (13.7% depression, 17.4% anxiety, 1.4% psychotic disorders, 6.6% addictive disorders, 5.4% insomnia) was observed [26].

The etiology of the psychiatric sequelae of SARS-CoV-2 infection is multifactorial and includes direct effects of viral (CNS) infection, excessive immune response, social isolation, uncertainty about the course of the disease, persistent symptom manifestation, and concern about recurrence of symptom exacerbation a.o. [23, 27, 28]. Studies suggest that immune system dysregulation can be associated with depressive symptoms [29] which may explain some of the psychiatric morbidity also following SARS-CoV-2 infection. As a correlate of COVID-19, imaging studies have found changes in the limbic system and related areas (prefrontal, anterior cingulate, and insular cortex). An abnormal functional disconnectivity may also play an important role [30]. These structural brain changes may lead to a link between COVID-19 and its psychopathological consequences in the long-term course of the disease.

Commonly known tools such as the Patient Health Questionaire (PHQ), the State and Trait Anxiety Index (STAI), the PTSD Checklist (PCLC), and the Fatigue Assessment Scale (FAS) are used frequently for symptom classification of post-COVID. To differentiate a depressive disorder from fatigue, it is recommended to ask about core depressive symptoms according to DSM-5 or ICD-11. Cognitive and memory impairment, attention, and executive function can be assessed by using neuropsychological test batteries [31, 32].

The current German S1 guideline for post-COVID/long-COVID primarily recommends multimodal treatment approaches [33]. Recent literature provides evidence for the anti-inflammatory and antiviral properties of various antidepressants, particularly selective serotonin reuptake inhibitors (SSRIs) in the acute phase of infection [34]. However, despite the large number of patients affected, there is no report on the efficacy of pharmacological treatment of post-COVID. Comorbid psychiatric disorders (depression, anxiety disorders, PTSD) should therefore be treated according to the current guidelines. Serotonin and norepinephrine reuptake inhibitors (SNRIs) with pain-modulating effects may be considered for additional pain symptomatology. Psychotherapy and cognitive training as well as measures to strengthen protective factors such as social support, and stress coping strategies should be used primarily. Since the cognitive impairments in post-COVID mainly affect planning thinking, concentration, memory, and language skills targeted cognitive training methods and programs can be attempted [8]. The first specific group psychotherapy programs have been developed, including knowledge transfer of post-COVID, mindfulness exercises, cognitive restructuring, and management of depressive moods, pain, and physical complaints [8]. However, in order to determine treatment success, such therapy programs should first be evaluated, for example, by neurocognitive pre- and posttests.

## Consequences

Looking at the literature in detail, it can be stated that the current data on post-COVID do not yet allow concrete conclusions for clinical care: (a) Due to the lack of specific diagnostics of post-COVID (missing post-COVID specific screening measures and biomarkers), differentiation from other diseases associated with similar symptoms, such as fatigue syndrome, intensive care syndrome, or depression due to contact prohibition, remains difficult [32, 35]. (b) Post-COVID may also generate novel complex and subjective symptoms, although it remains unclear to what extent the nature and duration of these symptoms correspond to the signs of other severe infectious diseases and whether established therapeutic approaches are applicable [36]. (c) In addition, causes, course and duration, and predictors of post-COVID syndrome, particularly in its severe form, have not been adequately specified yet. Further research is needed in this area, especially with regard to the long-term recording of symptoms and the frequency of complex post-COVID cases. (d) Finally, there is a lack of holistic care structures and treatments for this unclear but quite complex illness. The organizational structures, treatment strategies, the human and material resources needed in the long term to adequately treat patients with severe post-COVID, and the impact of care delivery on disease progression, remain unclear. However, studies on implementation and evaluation of interdisciplinary and multisectoral health and research networks for evidence-based treatment of patients with severe post-COVID syndrome are already underway, which will help us to better understand this complex clinical picture [8, 37].

## Future Perspective

Post-COVID highlights the link and transition between brain diseases and mental health. Accordingly, the post-COVID syndrome exemplifies the need for clinical, research, and teaching collaborations between neurology, psychiatry, infectious diseases, and others. Understanding and managing long-term neurological and psychiatric sequelae after COVID-19 will require additional common research investments and health care resources. Patient-centered services for post-COVID care require a proper definition of patient tracks from the primary care physician to specialized care. This should be built up on existing infrastructure on a level-based approach. Furthermore, longitudinal follow-up with phenotyping (clinical, biomarker, genetics, among others) is needed to better understand the global burden of post-COVID syndrome on a regional, community, and global level. Interdisciplinary longitudinal care needs to be accompanied by research to further understand disease mechanisms, risk factors, and prognosis. Federal funding initiatives designed to support a deeper comprehensive understanding of post-COVID syndrome are strongly needed. This will help to phenotype acute and post-COVID for specific treatment trials.

Common agenda for clinical care and research:

1. Research to better understand the neurobiological and other determinants of post-COVID syndrome including the impact of the different SARS-CoV-2 variants on incidence and phenomenology to identify the risk of an unrestricted ongoing SARS-CoV-2 infection for the society.
2. Identification of specific phenoytpes and biomarkers of post-COVID syndrome to improve prediction, prevention, diagnosis, and treatment.
3. Validation of new technologies (patient apps, smartwatches, Internet-based psychological interventions) for early recognition and care of post-COVID patients.
4. Development of international and multidisciplinary recommendations/guidelines for the diagnosis and treatment of post-COVID syndrome.
5. Cross-sectoral and interdisciplinary care concept for post-COVID integrating tailored prevention, pharmacotherapies, and rehabilitation.
6. Regular evaluation of the newly developed care structures, patient pathways, and the effects of interdisciplinary treatment strategies on the course of the disease.
7. Interdisciplinary clinical and research interactions on a national and international level.

## Conclusion

While most people with COVID-19 recover completely, a substantial number of patients experience prolonged symptoms. Addressing the patient’s needs of post-COVID syndrome requires a significant investment in existing resources and funding. The EAN and EPA join forces by organizing regular meetings of the “post-COVID working groups.” It is planned to combine data from longitudinal cohorts from both organizations to establish predictive data sets to identify individuals at risk for developing post-COVID syndrome. In addition, clinical trials are underway to develop evidence-based treatments of post-COVID mental and neurological syndromes. Special attention is being paid to the cognitive outcome as they form the basis of unfavorable outcomes in a substantial proportion of patients with post-COVID syndrome.
